# First human infection with *Onchocerca takaokai* (Spirurida: Onchocercidae) presenting as creeping eruption in Japan

**DOI:** 10.1051/parasite/2026023

**Published:** 2026-04-17

**Authors:** Eri Ikenaga, Eiji Nagayasu, Hideo Hasegawa, Hiroyuki Murota, Yutaka Kuwatsuka

**Affiliations:** 1 Department of Dermatology, Nagasaki University Hospital 1-7-1 Sakamoto Nagasaki Nagasaki 852-8501 Japan; 2 Department of Dermatology, Sasebo City General Hospital 9-3 Hirase-machi Sasebo Nagasaki 857-8511 Japan; 3 Division of Parasitology, Department of Infectious Diseases, Faculty of Medicine, University of Miyazaki 5200 Kiyotakecho Kihara Miyazaki Miyazaki 889-1692 Japan; 4 Department of Biomedicine, Faculty of Medicine, Oita University 1-1 Idaigaoka Hasama, Yufu Oita 879-5593 Japan

**Keywords:** *Onchocerca takaokai*, Creeping eruption, Zoonotic onchocerciasis, Human infection, Wild boar, Japan

## Abstract

Zoonotic onchocerciasis is a rare human infection caused by *Onchocerca* species that normally parasitize non-human mammals. In Japan, all previously reported human cases have been attributed to *Onchocerca japonica* and have presented as localized, non-migratory subcutaneous nodules. Here, we report the first human infection caused by *Onchocerca takaokai* Uni *et al.*, 2015. A 24-year-old male presented with linear migratory erythema on the forearm, clinically consistent with creeping eruption. Histopathological examination revealed an adult female filarial nematode with polymyarian-coelomyarian musculature, without internal cuticular ridges in the lateral cords, and lacking transverse ridges on the cuticle. Molecular analyses of the mitochondrial 12S rRNA and cytochrome c oxidase subunit I (cox1) genes confirmed that the parasite was *O. takaokai*, a parasite of wild boars in Japan. This case demonstrates a clinical presentation distinct from that of *O. japonica* and suggests that *O. takaokai* should be considered in the differential diagnosis of creeping eruption in endemic areas.

## Abstract (fr)

Première infection humaine par *Onchocerca takaokai* (Spirurida: Onchocercidae) se présentant sous forme d’éruption rampante au Japon. L’onchocercose zoonotique est une infection humaine rare causée par des espèces d’*Onchocerca* qui parasitent normalement les mammifères non humains. Au Japon, tous les cas humains rapportés jusqu’à présent ont été attribués à *Onchocerca japonica* et se sont manifestés par des nodules sous-cutanés localisés et non migrateurs. Nous rapportons ici le premier cas d’infection humaine à *Onchocerca takaokai* Uni *et al.*, 2015. Un homme de 24 ans présentait un érythème linéaire migrant sur l’avant-bras, cliniquement compatible avec une éruption rampante. L’examen histopathologique a révélé un nématode filaire femelle adulte à musculature polymyarienne-coelomyarienne, sans crêtes cuticulaires internes dans les cordons latéraux et sans crêtes transversales sur la cuticule. L’analyse moléculaire des gènes de l’ARNr 12S mitochondrial et de la sous-unité I de la cytochrome c oxydase (cox1) a confirmé qu’il s’agissait d’*O. takaokai*, un parasite du sanglier au Japon. Ce cas présente un tableau clinique différent de celui d’*O. japonica* et suggère qu’*O. takaokai* doit être pris en compte dans le diagnostic différentiel des éruptions cutanées rampantes en zones endémiques.

## Introduction

Zoonotic onchocerciasis is a rare parasitic disease caused by *Onchocerca* species that normally parasitize non-human mammals, such as wild ungulates, canids, or equids [4, 11, 12]. According to a 2020 review, since the first human case was recorded in 1965, a total of 40 cases have been documented worldwide, all within the Holarctic region [4]. To date, five species have been identified as causative agents of human zoonotic onchocerciasis, each primarily parasitizing specific ungulate or carnivore hosts: *O. lupi* (dogs; distributed in the USA, Europe, Türkiye, Tunisia, and Iran), *O. japonica* (formerly *O. dewittei japonica*, wild boars; Japan), *O. gutturosa* (cattle; Europe, North America, Africa, and Australia), *O. jakutensis* (red deer; Europe), and *O. cervicalis* (horses; North America and Europe) [4].

In Japan, the diversity of the genus *Onchocerca* is remarkably high. To date, 10 species have been recorded from domestic and wild ungulates: *O. gutturosa* and *O. lienalis* in cattle; *O. cervicalis* in horses; *O. suzukii*, *O. caprini* (originally *Loxodontofilaria caprini*), and *O. skrjabini* in Japanese serows; *O. eberhardi*, *O. flexuosa*, and *O. skrjabini* in sika deer; and *O. japonica* (originally *O. dewittei japonica*) and *O. takaokai* in wild boars [10, 14, 19, 21]. Despite this diversity, 13 documented cases of zoonotic onchocerciasis have been reported in Japan to date, all attributed to *O. japonica*, and were described as presenting with a single, localized subcutaneous nodule [7, 10].

Here, we report the first human case of *Onchocerca takaokai* Uni *et al.*, 2015 infection. Notably, the infection manifested as creeping eruption rather than a localized subcutaneous nodule, representing a clinical presentation fundamentally different from that observed in previously reported human cases caused by *O. japonica*.

## Materials and methods

### Ethics statement

This study was conducted in accordance with the Declaration of Helsinki. The institutional review board of Nagasaki University Hospital determined that formal ethical approval was not required for this single case report, and written informed consent for publication was obtained from the patient.

### Case description

A 24-year-old male noticed migratory erythema extending from the left upper arm to the forearm two months before presentation. The patient resided in Nagasaki Prefecture, Japan, in a suburban area adjacent to low mountains where the Japanese wild boar (*Sus scrofa leucomystax*) is occasionally observed. He had no history of travel abroad. He worked as an office employee and did not own any pets. Although he did not recall any blackfly bites, exposure could not be excluded. Despite treatment with topical steroids and oral antihistamines, the lesions persisted.

**Figure 1:**
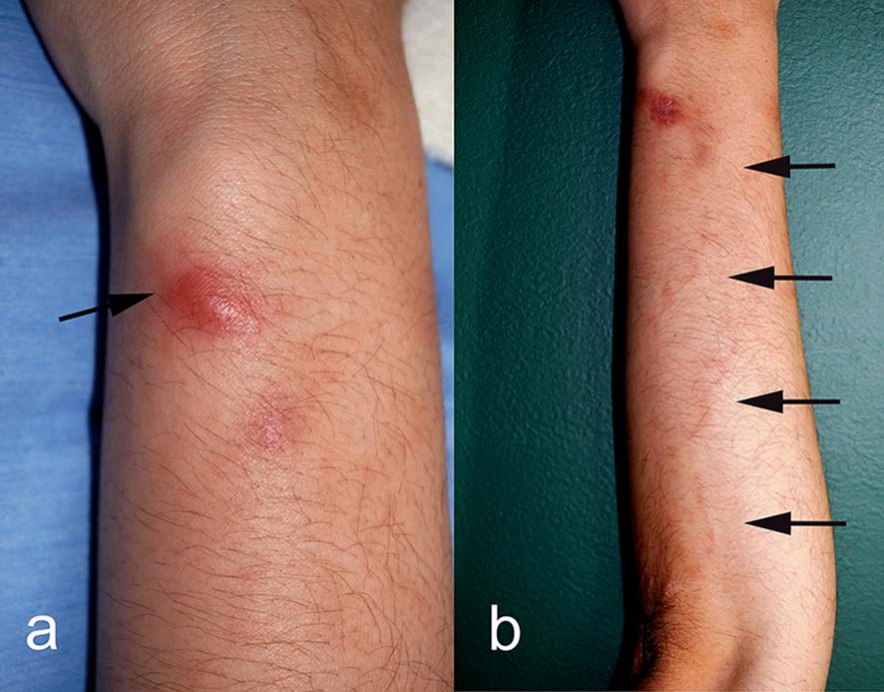
Clinical features. (a) Elastic reddish nodule on the extensor side of the left forearm (arrow). Skin biopsy was performed from this nodule. (b) Irregular, serpiginous erythematous streaks and eruptions, continuous with the nodule, were observed from the forearm to the radial side of the elbow, suggestive of creeping eruption (arrows).

At the first visit, a firm, elastic reddish nodule, approximately 1 cm in diameter, was observed on the extensor side of the left forearm (Fig. 1a), accompanied by an irregular, serpiginous linear erythematous eruption to the radial side of the elbow (Fig. 1b). Based on the clinical presentation, creeping disease was initially suspected, although the patient had no history of consuming raw freshwater fish. A skin biopsy revealed cross sections of a nematode consistent with a filarial parasite, and zoonotic filariasis was therefore suspected.

The patient was treated with oral ivermectin (12 mg) administered twice at a one-week interval, resulting in complete resolution within one month. No recurrence was observed at six-month follow-up.

### Histopathological examination

Skin biopsy specimens were fixed in formalin and embedded in paraffin. Histological sections were stained with hematoxylin and eosin and examined by light microscopy.

### Molecular analysis and phylogenetic analysis

Genomic DNA was extracted from formalin-fixed, paraffin-embedded tissue using DEXPAT (Takara Bio Inc., Kusatsu, Shiga, Japan). A partial region of the mitochondrial 12S rRNA gene was amplified by PCR using primers Diro12S-F and Diro12S-R, as previously described [13]. In addition, a portion of the mitochondrial cytochrome c oxidase subunit I (cox1) gene was amplified using the previously reported primers Fil_COX1F and Fil_COX1R [17]. PCR products were purified and sequenced directly. The newly obtained sequences were deposited in GenBank under accession numbers PV388830 (12S rRNA) and PZ054045 (cox1).

The obtained sequences were compared with sequences of *Onchocerca* species deposited in GenBank. A maximum likelihood phylogenetic tree based on the 12S rRNA gene was constructed using MEGA11 [16]. Pairwise *p*-distances (uncorrected) for the cox1 sequences were calculated based on a 347-bp alignment in Geneious Prime 2025.0.3 (https://www.geneious.com).

## Results and discussion

### Histopathological findings

**Figure 2:**
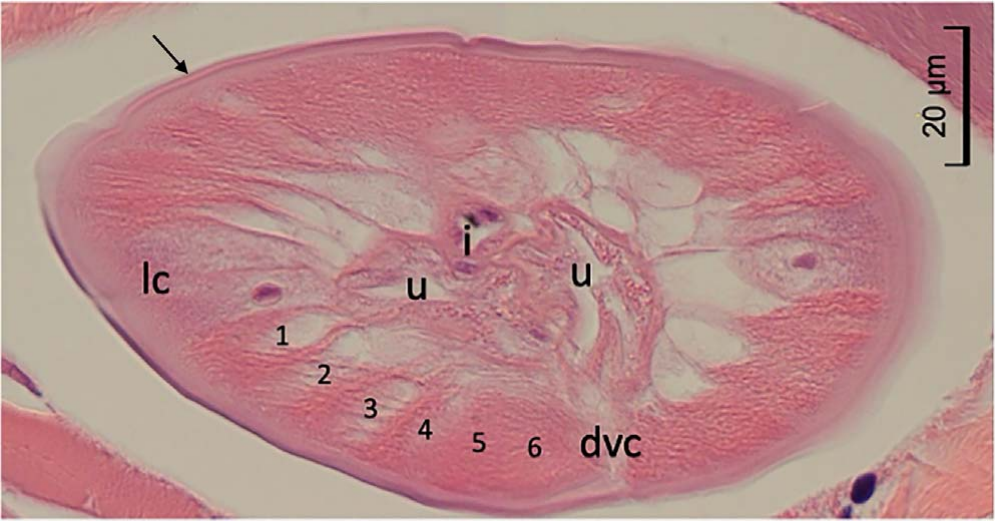
Histopathological examination. A cross section, cut slightly obliquely, of the worm found in the dermis of the patient, showing smooth cuticle (arrow), six somatic muscle cells (1–6) in a quadrant, lateral cord (lc) without internal cuticular projection, dorsal or ventral cord (dvc), and sections of intestine (i) and two uterine tubes (u) in the pseudocoelom.

Histological examination revealed a nematode cross section approximately 70 μm in diameter, surrounded by a 2 μm thick cuticle. Transverse striations were observed at approximately 1 μm intervals on the cuticle surface. Transverse ridges were absent on the cuticle. The somatic musculature was well developed and exhibited a polymyarian-coelomyarian pattern with six muscle cells per quadrant (Fig. 2). Sections of the intestine and two uterine tubes were observed in the pseudocoelom. No bacillary band was observed and the lateral cords were triangular or rectangular, without internal cuticular ridges. The presence of the uterine structures indicated that the nematode was in its fifth (adult) stage.

To differentiate this nematode from other known causes of creeping disease, a detailed morphological comparison was performed. Among the nematodes known to cause creeping disease in Japan that possess a polymyarian-coelomyarian musculature are *Gnathostoma* spp., *Crassicauda giliakiana* (also known as the larval spirurid-type X), and zoonotic filariae [1, 5]. However, *Gnathostoma* species possess spines on their cuticle, and their musculature exhibits an intermediate pattern between the polymyarian-coelomyarian and meromyarian-platymyarian types, differing from the present case [1, 5]. Similarly, *C. giliakiana* has a significantly higher number of muscle cells and remains in the larval stage without developing reproductive organs in human hosts, distinguishing it from the present nematode [3, 9]. Conversely, zoonotic filariae lack cuticular spines and often reach adulthood in humans. Zoonotic filarial species previously identified in the subcutaneous connective tissues of humans in Japan include *Dirofilaria immitis*, *Dirofilaria repens*, *Dirofilaria ursi*, and *Onchocerca japonica* [2, 13, 15, 22]. *Dirofilaria* species exhibit well-developed internal cuticular ridges [8], being readily distinguished from the present worm. Meanwhile, the relatively small number of somatic muscle cells in the sectioned worm suggests affinity with *Onchocerca* [11]. Although many species of *Onchocerca*, such as *O. japonica*, possess transverse ridges on the female cuticle [18], some congeners lack these structures. In Japan, two species – *O. suzukii*, parasitic in the Japanese serow, and *O. takaokai*, described from the Japanese wild boar – are known to lack transverse cuticular ridges [19, 21]. Because comparative cross-sectional morphological data for these species remain limited, definitive species identification was deferred until molecular analysis was performed.

### Molecular identification

**Figure 3:**
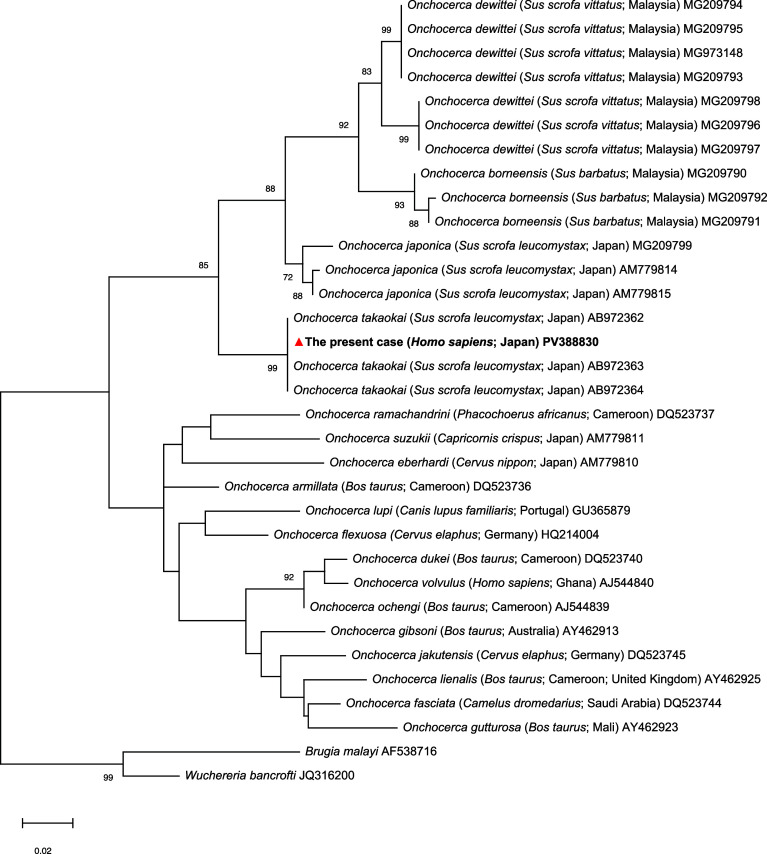
Phylogenetic placement of the *Onchocerca* species from the present case in Japan based on mitochondrial 12S rRNA gene sequences. The tree was constructed using the maximum likelihood method with the Hasegawa-Kishino-Yano model. Evolutionary rate differences among sites were modeled using a discrete Gamma distribution. The final dataset contained 33 sequences with a total of 377 aligned positions. Bootstrap values (≥70%) based on 1,000 replicates are shown next to the branches. The tree was rooted using *Brugia malayi* and *Wuchereria bancrofti* as outgroups. The present case (*Homo sapiens*, Japan) is indicated by a triangle (▲). Host species and geographic origin are shown in parentheses. All analyses were performed using MEGA11 (Tamura *et al.*, 2021) [16]. Scale bar indicates the number of substitutions per site.

Phylogenetic analysis based on mitochondrial 12S rRNA gene sequences demonstrated that the sequence obtained from the present case clustered with *O. takaokai* isolates from wild boars in Japan, with high bootstrap support (Fig. 3). To further confirm species identity, pairwise *p*-distances were calculated based on a 347-bp alignment of the mitochondrial cytochrome c oxidase subunit I (cox1) gene. The present isolate showed no genetic divergence (*p*-distance = 0.000) from all three reference sequences registered as *O. takaokai*. In contrast, the genetic divergence between the present isolate and *O. suzukii* was 0.098, while that between the present isolate and *O. japonica* ranged from 0.072 to 0.078 (Table S1).

### Clinical and epidemiological implications

Taken together, the morphological and molecular findings indicate that the parasite responsible for the present infection was *O. takaokai*. The species *O. takaokai* was first described in 2015 as a second species parasitizing the Japanese wild boar (*Sus scrofa leucomystax*) [19]. Recent epidemiologic studies have revealed its geographical distribution in southwestern Japan; *O. takaokai* has been reported from wild boars in Kyushu (Oita Prefecture), with a prevalence of approximately 26.9%, and from the Ryukyu wild boar (*S. s. riukiuanus*) on Kakeroma Island in the Nansei Islands [20]. Both *O. japonica* and *O. takaokai* share a common natural vector, the anthropophilic and zoophilic blackfly *Simulium bidentatum* [6, 19]. However, despite its recognized presence in wild boars and its transmission by a human-biting blackfly, no human infections attributable to *O. takaokai* had been reported prior to the present case.

The present case represents the first documented human infection caused by *O. takaokai*. Consequently, the number of *Onchocerca* species confirmed to infect humans increases from five to six, and the clinical spectrum of zoonotic onchocerciasis in Japan is expanded. A notable clinical feature was creeping eruption, in contrast to previously reported human infections caused by *O. japonica*, which have consistently presented as localized, non-migratory subcutaneous nodules [7, 10].

Zoonotic onchocerciasis is not routinely considered in patients presenting with creeping eruption; however, this case suggests that *O. takaokai* should be included in the differential diagnosis in regions where the parasite has been reported in wild boars. The occurrence of this case in Nagasaki Prefecture, Kyushu, is epidemiologically plausible, as *O. takaokai* has been documented in wild boars on the same island. However, given that wild boars are widely distributed throughout Japan and the vector blackfly *Simulium bidentatum* also occurs broadly, the actual distribution of *O. takaokai* may be wider than currently recognized [20]. Molecular identification remains essential for accurate species determination and for improving understanding of the clinical diversity of zoonotic *Onchocerca* infections in humans.

## Supplementary material

*Table S1*. Pairwise p-distances based on mitochondrial cox1 sequences between the present isolate and related *Onchocerca* species. The supplementary material of this article is available at https://www.parasite-journal.org/10.1051/parasite/2026023/olm.

## References

[1] Akahane H. 2003. Gnathostomiasis (1) *Gnathostoma spinigerum* and *G. doloresi*, in Progress of Medical Parasitology in Japan, Otsuru M, Kamegai S, Hayashi S, Editors. Meguro Parasitological Museum: Tokyo, Japan. p. 485–505.

[2] Akao N. 2011. Human dirofilariasis in Japan. Tropical Medicine and Health, 39(1 Suppl 2), 65–71. 22028604 10.2149/tmh.39-1-suppl_2-65 PMC3153151

[3] Ando K, Inaba T, Sato Y, Miura K, Chinzei Y. 1992. Morphological features in cross section of larva of the suborder Spirurina (Nematoda) suspected as the causative agent of creeping eruption. Japanese Journal of Parasitology, 41, 46–48.

[4] Cambra-Pelleja M, Gandasegui J, Balana-Fouce R, Munoz J, Martinez-Valladares M. 2020. Zoonotic implications of *Onchocerca* species on human health. Pathogens, 9(9), 761. 32957647 10.3390/pathogens9090761 PMC7560048

[5] Chitwood M, Lichtenfels JR. 1972. Identification of parasitic metazoa in tissue sections. Experimental Parasitology, 32(3), 407–519. 4675138 10.1016/0014-4894(72)90069-0

[6] Fukuda M, Otsuka Y, Uni S, Bain O, Takaoka H. 2010. Molecular identification of infective larvae of three species of *Onchocerca* found in wild-caught females of *Simulium bidentatum* in Japan. Parasite, 17(1), 39–45. 20387737 10.1051/parasite/2010171039

[7] Fukuda M, Uni S, Igari T, Utsumi Y, Otsuka Y, Nakatani J, Uga S, Hara T, Hasegawa H, Takaoka H. 2019. Human case of *Onchocerca dewittei japonica* infection in Fukushima, Northeastern Honshu, Japan, Parasitology International, 72, 101943. 31220633 10.1016/j.parint.2019.101943

[8] Gutierrez Y. 2000. Cutaneous larva migrans, in Diagnostic Pathology of Parasitic Infections with Clinical Correlations, 2nd ed. Oxford University Press: New York. p. 343–353.

[9] Hasegawa H. 2003. Larval spirurin infections, in Progress of Medical Parasitology in Japan, Otsuru M, Kamegai S, Hayashi S, Editors. Meguro Parasitological Museum: Tokyo, Japan. p. 519–528.

[10] Okazaki D, Fukuda M, Hebisawa A, Uni S, Junker K, Suzuki Y, Nakano M, Agatsuma T, Hasegawa H, Yamada M, Nakatani J, Hara T, Martin C, Kimura D, Takaoka H. 2022. Zoonotic infection caused by *Onchocerca japonica* (Nematoda: Filarioidea) in a 69-year-old woman in Kanto region, eastern Honshu, Japan, Parasitology International, 91, 102643. 35961578 10.1016/j.parint.2022.102643

[11] Orihel TC, Eberhard ML. 1998. Zoonotic filariasis. Clinical Microbiology Reviews, 11(2), 366–381. 9564568 10.1128/cmr.11.2.366 PMC106837

[12] Otranto D, Eberhard ML. 2011. Zoonotic helminths affecting the human eye. Parasites & Vectors, 4, 41. 21429191 10.1186/1756-3305-4-41 PMC3071329

[13] Suzuki J, Kobayashi S, Okata U, Matsuzaki H, Mori M, Chen KR, Iwata S. 2015. Molecular analysis of *Dirofilaria repens* removed from a subcutaneous nodule in a Japanese woman after a tour to Europe. Parasite, 22, 2. 25619827 10.1051/parasite/2015002 PMC4306022

[14] Takaoka H. 2015. Zoonotic onchocerciasis in Japan: its causative *Onchocerca* species and vector black fly species. Medical Entomology and Zoology, 66(2), 23–30. (in Japanese)

[15] Takaoka H, Bain O, Uni S, Korenaga M, Tada K, Ichikawa H, Otsuka Y, Eshita Y. 2001. Human infection with *Onchocerca dewittei japonica*, a parasite from wild boar in Oita, Japan. Parasite, 8(3), 261–263. 11584758

[16] Tamura K, Stecher G, Kumar S. 2021. MEGA11: molecular evolutionary genetics analysis version 11. Molecular Biology and Evolution, 38(7), 3022–3027. 33892491 10.1093/molbev/msab120 PMC8233496

[17] To KK, Wong SS, Poon RW, Trendell-Smith NJ, Ngan AH, Lam JW, Tang TH, AhChong AK, Kan JC, Chan KH, Yuen KY. 2012. A novel *Dirofilaria* species causing human and canine infections in Hong Kong. Journal of Clinical Microbiology, 50(11), 3534–3541. 22915604 10.1128/JCM.01590-12 PMC3486214

[18] Uni S, Bain O, Takaoka H, Miyashita M, Suzuki Y. 2001. *Onchocerca dewittei japonica* n. subsp., a common parasite from wild boar in Kyushu Island, Japan. Parasite, 8(3), 215–222. 11584751 10.1051/parasite/2001083215

[19] Uni S, Fukuda M, Agatsuma T, Bain O, Otsuka Y, Nakatani J, Matsubayashi M, Harada M, Omar H, Ramli R, Hashim R, Azirun MS, Takaoka H. 2015. *Onchocerca takaokai* n. sp. (Nematoda: Filarioidea) in Japanese wild boars (*Sus scrofa leucomystax*): description and molecular identification of intradermal females. Parasitology International, 64(6), 493–502. 26165205 10.1016/j.parint.2015.07.001

[20] Uni S, Fukuda M, Uga S, Agatsuma T, Nakatani J, Suzuki K, Yokohata Y, Kimura D, Takaoka H. 2021. Prevalence of *Onchocerca japonica* and *O. takaokai* infections in the Japanese wild boar, *Sus scrofa leucomystax*, and the Ryukyu wild boar, *S. s. riukiuanus*, in Japan. Parasitology International, 83, 102313. 33662527 10.1016/j.parint.2021.102313

[21] Yagi K, Bain O, Shoho C. 1994. *Onchocerca suzukii* n. sp. and *O. skrjabini* (= *O. tarsicola*) from a relict bovid, *Capricornis crispus*, in Japan. Parasite, 1(4), 349–356. 9140501 10.1051/parasite/1994014349

[22] Yamada M, Shishito N, Nozawa Y, Uni S, Nishioka K, Nakaya T. 2017. A combined human case of *Dirofilaria ursi* infection in dorsal subcutaneous tissue and *Anisakis simplex sensu stricto* (s.s.) infection in ventral subcutaneous tissue. Tropical Medicine and Health, 45, 26. 10.1186/s41182-017-0067-4 PMC5664901 29118653

